# The Role of Papaverine in the Molecular and Biomechanical Mechanisms of Cervical Ripening: A Narrative Review

**DOI:** 10.3390/ijms27146344

**Published:** 2026-07-17

**Authors:** Wojciech Flis, Mateusz Wartęga, Tomasz Góra, Maciej W. Socha

**Affiliations:** 1 Department of Perinatology, Gynecology and Gynecologic Oncology, Faculty of Health Sciences, Collegium Medicum in Bydgoszcz, Nicolaus Copernicus University, Łukasiewicza 1, 85-821 Bydgoszcz, Poland; 2 Department of Obstetrics and Gynecology, St. Adalbert’s Hospital in Gdańsk, Copernicus Healthcare Entity, Jana Pawła II 50, 80-462 Gdańsk, Poland; 3 Department of Pathophysiology, Faculty of Pharmacy, Collegium Medicum in Bydgoszcz, Nicolaus Copernicus University, M. Curie-Skłodowskiej 9, 85-094 Bydgoszcz, Poland; 4 Department of Gynecology and Obstetrics, John Paul II City Hospital, Rycerska 4, 35-241 Rzeszów, Poland

**Keywords:** papaverine, cervical ripening, induction of labor, phosphodiesterase inhibitor, inflammation, nitric oxide, cervical biomechanics

## Abstract

Papaverine (PPV) has long been used in obstetric practice to facilitate cervical dilatation, yet its mechanism of action remains poorly understood. This creates an apparent paradox, as experimental studies indicate that PPV suppresses several inflammatory signaling pathways traditionally considered essential for physiological cervical ripening, despite its reported clinical benefits during labor. In this narrative review, we critically integrate current knowledge of cervical ripening biology with the pharmacological actions of PPV to reconcile this discrepancy. We propose that the reported clinical effects of PPV may be explained predominantly by modulation of cervical biomechanics rather than by direct enhancement of inflammatory remodeling. In particular, smooth muscle relaxation and cyclic nucleotide-dependent signaling may reduce functional cervical resistance and facilitate cervical dilatation despite the predominantly anti-inflammatory molecular profile of PPV. Although this mechanistic framework remains hypothetical, it provides a biologically plausible explanation for the apparent discrepancy between the molecular and clinical effects of papaverine and highlights priorities for future experimental and clinical research.

## 1. Introduction

The uterine cervix remains one of the most enigmatic and dynamic structures in reproductive biology. Despite its relatively small size, it plays a central role in maintaining pregnancy and orchestrating the transition to labor. During most of gestation, the cervix functions as a firm mechanical barrier that preserves uterine integrity and protects the intrauterine environment. In contrast, at the onset of labor it undergoes profound biochemical, structural, and biomechanical remodeling that transforms it into a compliant and distensible tissue capable of allowing fetal passage through the birth canal. This process, commonly referred to as cervical ripening, involves highly coordinated alterations in extracellular matrix organization (ECM), collagen architecture, tissue hydration, inflammatory signaling, and smooth muscle function [1]. Cervical ripening is now widely recognized as a sterile inflammatory process regulated by complex interactions between resident cervical cells, infiltrating leukocytes, cytokines, prostaglandins, nitric oxide, and multiple intracellular signaling pathways. The growing understanding of these pathways has significantly expanded the modern concept of labor as an inflammatory-like physiological event [2,3].

PPV, an opium alkaloid and non-selective phosphodiesterase inhibitor, has long been used clinically as a smooth muscle relaxant in various medical fields, including obstetrics [4,5]. Several clinical studies suggest that PPV administration may shorten the duration of labor and facilitate cervical dilatation [6]. However, its precise mechanism of action in the context of cervical ripening remains poorly understood. Interestingly, experimental and molecular studies indicate that PPV may suppress several inflammatory signaling pathways involved in cervical remodeling, including cytokine production, oxidative stress responses, and other pro-inflammatory cascades [7]. These observations appear partially inconsistent with the contemporary inflammatory paradigm of cervical ripening [8]. This apparent discrepancy raises an important mechanistic question: does PPV truly accelerate biochemical cervical ripening, or does it primarily facilitate labor through functional relaxation and reduction in cervical resistance independent of classical inflammatory remodeling? Addressing this question may improve our understanding of both cervical physiology and the pharmacological modulation of labor progression.

Therefore, the aim of this review is not only to summarize the current understanding of the inflammatory and molecular mechanisms underlying cervical ripening, but also to critically evaluate how the known pharmacological actions of PPV intersect with these pathways. Unlike previous reviews, which have generally focused either on the biology of cervical ripening or on the pharmacological properties of PPV separately, this review integrates both fields to address the apparent paradox between the predominantly anti-inflammatory molecular profile of PPV and the reported clinical effects on cervical dilatation. Based on the available evidence, we propose a hypothesis-generating mechanistic framework suggesting that PPV may facilitate labor predominantly through biomechanical and functional mechanisms rather than by directly promoting inflammatory cervical remodeling. Specifically, we hypothesize that the apparent clinical efficacy of PPV may result predominantly from modulation of cervical biomechanics and smooth muscle function rather than from direct enhancement of inflammatory ECM remodeling.

## 2. Structural and Biomechanical Basis of Cervical Ripening

The cervix, which is the lower part of the uterus, consists of a vaginal and supravaginal portion. The cervical canal runs through the center of the cervix and is lined with cylindrical epithelium (which consists of a single layer of epithelial cells) [9]. The epithelial cells are primarily responsible for the production of thick mucus, which during pregnancy tightly fills the cervical canal, creating an immunobiological and mechanical barrier maintaining physical integrity throughout pregnancy. The mucus produced by the epithelial cells is rich in inorganic ions, enzymes, proteins, and mucin glycoproteins. As gestational age progresses, the epithelial cells of the cervical canal proliferate intensively, which leads to increased production of cervical mucus [10].

From a histological point of view, the cervix is composed mainly of connective tissue, ground substance, smooth muscle fibers, and a cellular component [11]. These components are infiltrated by a number of blood vessels, nerves and lymphatic vessels. The cellular compartment comprises fibroblasts, wandering cells, mast cells and neutrophils (especially during local inflammatory response) [12]. Smooth muscle fibers constitute a small percentage of the cervical matrix, undergoing different spatial organization depending on the location—in the area of the internal cervical os they form a more organized structure. In turn, in the external cervical os area, muscle fibers are more loosely dispersed in the space [13]. The primary component of the dry mass of the cervix is its ECM also known as the cervical ground substance. The basic building block of cervical ECM is composed mainly of collagen type I and type III, with a small percentage of collagen IV fibers. Fibroblasts, which actively participate in the metabolism of cervical tissue, are responsible for maintaining collagen homeostasis. Interestingly, during cervical ripening, a significant decrease in type I collagen mRNA expression is observed in the cervical stroma [14]. The collagen fibers of the cervical stroma form an organized spatial structure by forming numerous covalent bonds between themselves. The formation of strong covalent bonds enables the creation of a degradation-resistant three-dimensional structure, which ensures the stiffness of the whole cervical tissue during pregnancy [15]. Additionally, cervical ECM is composed of glycosaminoglycans (GAGs) which contain a vast amount of free sulfate groups which makes them highly hydrophilic molecules [16]. The main GAGs in the cervical ECM are: dermatan sulfate, chondroitin sulfate, heparan sulfate (HS) and hyaluronan (HA) [17,18,19]. Like collagen fibers, GAGs have the ability to form covalent bonds with the protein core, leading to the formation of highly ramified molecules—proteoglycans [20]. Subsequently, proteoglycans can bind free collagen fibers via their free anionic residues. The formation of such unique three-dimensional structure enables the creation of a compact, firm structure which is resistant to proteolytic degradation [16,20,21]. Taking the above into account, it seems obvious that qualitative (as well as quantitative) changes in proteoglycans and collagen spatial formation may have a profound impact on the biomechanics of cervical tissue and may also be crucial in the context of cervical ripening.

The last but not least of the elements that compose the cervical stroma are elastin fibers [22]. Individual elastin fibers form elastin polymers, which are then cross-linked into more advanced spatial structures known as microfibrils [23,24]. Changes in the spatial arrangement of microfibrils remain an interesting issue. In the non-pregnant state, microfibrils are arranged in long bundles that form a well-organized, tight structure. During pregnancy, the structure of elastic fibers becomes less organized and microfibrils become more randomly dispersed in the cervical tissue [22,23,24]. This spatial change in structure during pregnancy may suggest a possible involvement of steroid hormones in elastin fiber metabolism.

As mentioned earlier, in the perinatal period, the cervical tissue undergoes a thorough spatial rearrangement. These changes are a derivative of enzymatic degradation, development of a local inflammatory reaction, apoptosis and endocrine changes accompanying pregnancy [12]. These events subsequently lead to a significant loosening of the previously tightly packed cervical tissue and a great increase in the softness of the lower portion of the cervix. These biochemical events can be summarized as total spatial rearrangement of cervical ECM. The main histological and biochemical changes observed in the cervix are degradation of collagen and elastin fibers (accompanied by decrease in total collagen concentration), increased synthesis of HA, increased water influx, increased cervical cell apoptosis and infiltration of inflammatory cells that mediate the development of local inflammatory response [18,25,26]. The entire process of cervical ripening is a wide range of interdependent (and interrelated) molecular and biochemical pathways that are tightly controlled by a variety of regulatory factors [2,27].

The changes that are most pronounced during cervical ripening are a decrease in collagen content in the cervical ECM with a simultaneous increase in hyaluronan concentration [19,21,28,29]. At the end of gestation, HA becomes the dominant GAG of the cervical stroma. The high concentration of HA, which is a highly hydrophilic molecule, is responsible for increased water influx leading to the spatial dispersion of collagen fibers [18]. On the other hand, a decrease in collagen concentration is achieved mainly by reduced expression of collagen assembly genes and increased enzymatic degradation of collagen fibers [15].

Matrix metalloproteinases (MMPs), principally the gelatinases MMP-2 and MMP-9 together with the neutrophil collagenase MMP-8, are prime performers of cervical ripening due to their ability to directly cleave cervical collagen covalent bonds [30]. Additionally, MMPs can degrade other ECM elements such as elastin fibers, proteoglycans or fibronectin [31,32]. The observed increased influx and activation of inflammatory cells during peripartum period, particularly macrophages and neutrophils, results in enhanced local MMPs production. As term approaches, both MMPs expression and proteolytic activity rise markedly within the cervix and lower uterine segment [33,34]. The enzymatic activity and secretion of MMPs is tightly regulated by other factors involved in parturition. The expression of MMPs is enhanced by pro-inflammatory cytokines, nitric oxide (NO) or prostaglandins (PGs) [35]. In turn, activity of MMPs is suppressed mainly by tissue inhibitors of metalloproteinases (TIMPs) and progesterone (the concentration of which increases dramatically during pregnancy) [36,37]. The entire process of cervical ripening remains under the strict influence of a variety of molecular regulatory factors.

During cervical ripening, a local inflammatory reaction occurs. Increased vascular permeability leads to increased influx of water and inflammatory cells (such as neutrophils, mast cells, and macrophages). The presence of inflammatory cells leads to increased secretion of inflammatory mediators (such as prostaglandins, nitric oxide, pro-inflammatory cytokines and transcription factors), which play a key role in mediating the proper course of cervical ripening pathways [38,39].

**Figure 1:**
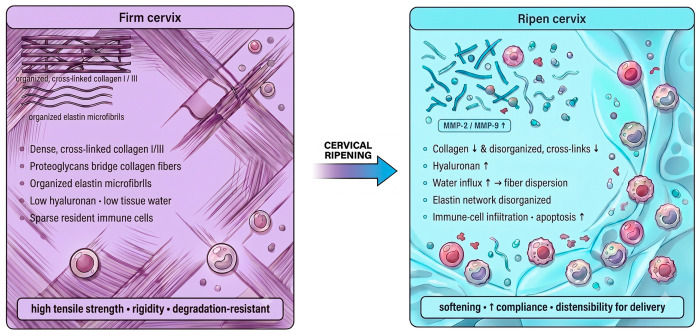
Schematic representation of the structural and cellular changes accompanying physiological cervical ripening. During the process of cervical ripening, the cervix undergoes progressive structural changes characterized by collagen fiber disorganization, degradation of collagen cross-links, increased hyaluronan content, disruption of the elastin network, enhanced tissue hydration, MMP-mediated ECM degradation, and infiltration of inflammatory cells. Collectively, these changes reduce tissue stiffness and increase cervical compliance, facilitating cervical dilatation during labor.

The uterine cervix represents a specialized and highly adaptable connective tissue structure whose biomechanical properties are largely determined by the organization and composition of its ECM. Throughout pregnancy and particularly during the peripartum period, the cervix undergoes extensive and tightly regulated structural remodeling that is essential for successful labor and delivery. Disturbances in this process may have important clinical consequences: accelerated cervical softening may contribute to cervical insufficiency and preterm birth, whereas delayed or inadequate remodeling can result in dysfunctional labor progression. Although considerable advances have been made in understanding cervical biology, many of the molecular mechanisms controlling cervical maturation remain incompletely characterized. Current evidence suggests that cervical ripening is regulated through a complex interaction between ECM remodeling, inflammatory mediators, vascular changes, and intracellular signaling pathways (Figure 1). Given the widespread obstetric use of PPV and its known effects on nucleotide signaling, smooth muscle relaxation, and vascular tone, it is plausible that this compound may influence one or more pathways involved in cervical remodeling. However, the potential relationship between PPV administration and cervical ripening remains largely unexplored.

## 3. Molecular Regulation of Cervical Ripening

Cervical ripening is currently recognized as a highly coordinated biological process regulated by a complex network of molecular and cellular signaling pathways rather than a passive phenomenon involving simple tissue softening. Increasing evidence suggests that cervical maturation shares many characteristics with a localized inflammatory response, in which mediators traditionally associated with immune activation participate directly in ECM remodeling and structural adaptation of cervical tissue [8]. This concept has substantially changed the understanding of cervical biology and emphasizes that cervical remodeling should be considered a tightly regulated immunobiological event [32].

At the tissue level, cervical ripening is associated with progressive infiltration of the cervical stroma by inflammatory cells, (including neutrophils, macrophages, mast cells, and wandering cells) [40]. The recruitment of these cells is promoted by dynamic alterations within the local microenvironment, including increased expression of chemokines, enhanced adhesion molecule activity, and elevated vascular permeability, which collectively facilitate cellular migration into the cervical stroma [41]. The biological significance of infiltrating inflammatory cells is largely related to their ability to produce and release a range of mediators involved in both tissue remodeling and amplification of local inflammatory activity. Activated macrophages, neutrophils, and fibroblasts contribute to ECM remodeling through the secretion of MMPs, which enzymatically degrade collagen fibers and other ECM components, leading to a reduction in cervical tissue integrity [39,42]. In parallel, these cells release numerous inflammatory mediators, including pro-inflammatory cytokines (such as IL-1, IL-6, tumor necrosis factor-α (TNF-α) and IL-8), together with PGs, NO, and reactive oxygen species (ROS) [43]. The coordinated action of these factors creates a self-reinforcing inflammatory microenvironment that promotes progressive cervical remodeling. In addition to their direct effects on ECM organization and tissue biomechanics, many of these mediators activate intracellular signaling pathways that coordinate the progression of cervical maturation [38,43,44,45].

**Figure 2:**
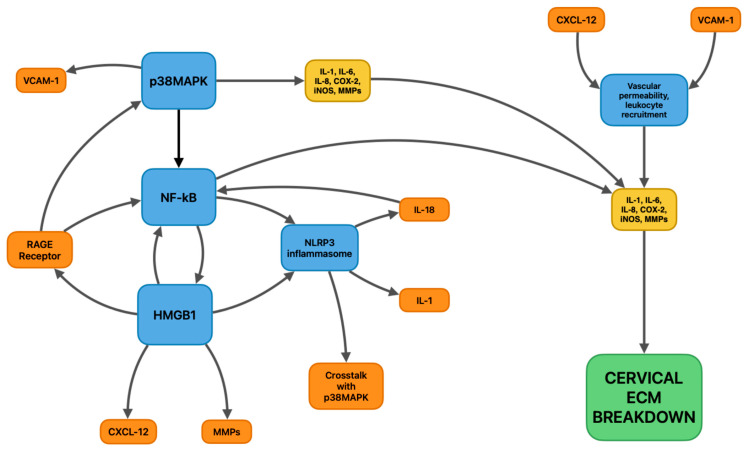
Schematic representation of the major molecular pathways involved in cervical ripening. Physiological cervical ripening is regulated by a complex network of interconnected inflammatory signaling pathways, including HMGB1/RAGE, NF-κB, p38 MAPK, and the NLRP3 inflammasome. These pathways collectively regulate the production of pro-inflammatory cytokines, chemokines, NO, MMPs, and adhesion molecules, thereby promoting leukocyte recruitment, increased vascular permeability, and ECM remodeling. The schematic illustrates the principal interactions between these pathways and is intended to provide a simplified overview of their coordinated contribution to cervical ripening.

Since substantial crosstalk exists among these pathways, cervical ripening should be considered a dynamic network of interrelated molecular events rather than a sequence of isolated biochemical mechanisms. Therefore, the following sections discuss the principal molecular regulators involved in cervical maturation, with particular emphasis on inflammatory cytokines, prostaglandins, nitric oxide signaling, inflammasome activation, and intracellular transcriptional mechanisms (Figure 2). The following sections provide a detailed overview of these mechanisms and their contribution to the regulation of cervical remodeling.

### 3.1. Interleukin-1

IL-1 appears to be a key cytokine involved in cervical ripening [46,47]. Studies have demonstrated that concentrations of IL-1 isoforms increase markedly in the cervicovaginal fluid of pregnant women during the peripartum period [47]. Moreover, intracervical administration of IL-1-containing suppositories has been shown to enhance the infiltration of neutrophils and increase chemokine levels within the cervical ECM [48]. These findings suggest that IL-1 may exert a multifaceted influence on the cervix, acting through both direct and indirect mechanisms to promote cervical remodeling.

Beyond its direct effects on cervical tissue, IL-1 regulates several downstream pathways that contribute to cervical remodeling. One of its major actions involves the modulation of prostaglandin homeostasis. IL-1 promotes prostaglandin accumulation by suppressing the activity of prostaglandin dehydrogenase (PGDH), the enzyme responsible for prostaglandin degradation, while concurrently upregulating cyclooxygenase-2 (COX-2), a critical enzyme in prostaglandin biosynthesis [49,50,51]. This shift toward increased PGs availability facilitates cervical softening and strengthens local inflammatory responses. Furthermore, IL-1 stimulates the production of additional pro-inflammatory mediators, such as IL-6 and IL-8, which enhance leukocyte infiltration and sustain the sterile inflammatory microenvironment within the cervix [46]. In contrast, the direct effects of IL-1 on cervical ripening are largely mediated through its regulation of ECM enzymatic cleavage. IL-1 promotes the expression and activity of MMPs while concurrently reducing the expression of TIMPs [46,52,53]. This shift in the MMP–TIMP balance favors enhanced collagen breakdown and increased proteolytic remodeling of the cervical ECM, contributing to the structural changes associated with cervical maturation. Through these interconnected mechanisms, IL-1 functions as a key mediator linking immune activation with ECM remodeling during cervical ripening.

### 3.2. Interleukin-8

IL-8 is widely recognized as a critical mediator of cervical ripening, acting at the interface of inflammatory signaling and ECM remodeling. Produced by a variety of immune cells (including macrophages, neutrophils, fibroblasts, and chorio-decidual cells), IL-8 expression increases substantially in cervical tissue toward term, with its local concentration closely correlating with the progression of cervical maturation [54,55].

One of the principal functions of IL-8 is the recruitment and activation of neutrophils within the cervical stroma. As a potent chemotactic factor, IL-8 promotes neutrophil migration into cervical tissue while simultaneously enhancing vascular permeability, thereby facilitating the influx of inflammatory cells [26,56]. The accumulation of activated neutrophils contributes directly to cervical remodeling through the release of MMPs (particularly MMP-8 and MMP-9) [57]. Moreover, IL-8 also possesses the ability to directly increase secretion and activation of MMPs, thereby facilitating enzymatic degradation of collagen covalent bonds within the cervical ECM [54].

Beyond its independent actions, IL-8 operates within a complex network of inflammatory mediators. Experimental evidence indicates that IL-1 enhances IL-8 production within cervical tissues, establishing a regulatory axis that amplifies local inflammatory response [58,59]. This interaction is further strengthened by PGs, whose synthesis is stimulated by IL-1. Notably, prostaglandin E_2_ has been shown to potentiate the chemotactic effects of IL-8 by lowering the threshold concentration required to induce neutrophil chemotaxis [46,60,61]. These observations suggest the existence of a coordinated cytokine–prostaglandin signaling loop that integrates immune activation with ECM remodeling during cervical ripening. The biological significance of IL-8 is further underscored by observational studies associating IL-8 with cervical dilation and leukocyte influx [57], while clinical evidence has identified IL-8 as a mediator of final cervical ripening in humans [55]. Collectively, these findings identify IL-8 as one of the pivotal regulators coordinating leukocyte recruitment, inflammatory activation, and ECM remodeling.

### 3.3. Prostaglandins

PGs are among the most powerful regulators of cervical ripening. Their role in labor induction is well established and supported by extensive experimental and clinical evidence, with prostaglandin analogs being widely used as effective pharmacological agents for cervical ripening [62,63,64]. The effects of PGs extend beyond their direct involvement in ECM remodeling. They also serve as key mediators that coordinate multiple inflammatory pathways, linking immune activation with the biochemical and structural changes required for cervical maturation [65].

The biological effects of prostaglandins are mediated through four G protein-coupled E-prostanoid (EP) receptors, designated EP1–EP4 [66]. Activation of these receptor subtypes elicits distinct cellular responses depending on their associated intracellular signaling pathways [67]. EP1 signaling is linked to calcium mobilization and promotes smooth muscle contraction, whereas EP2 and EP4 stimulate cyclic AMP (cAMP) production, resulting in smooth muscle relaxation [66,68]. In contrast, EP3 reduces intracellular cAMP levels and generally favors contractile activity [66]. EP receptor expression is dynamically regulated throughout pregnancy and varies across uterine tissues [67,69]. Differential expression of EP receptor subtypes near term is thought to facilitate the transition from pregnancy to labor by coordinating relaxation of the lower uterine segment and contractile activity of the upper uterus [70]. In addition to their well-established role in regulating myometrial activity, prostaglandins are important mediators of cervical ripening [67,71]. Current evidence indicates that prostaglandin-induced cervical remodeling is mediated predominantly through EP4 receptor signaling [72]. Notably, EP4 expression reaches its highest levels in cervical tissue at term, supporting the concept that activation of this receptor plays a central role in coordinating the biochemical and structural changes that characterize cervical maturation [67,72].

PGs play a pivotal role in coordinating the inflammatory and structural changes that characterize cervical ripening. Through interactions with pro-inflammatory cytokines, they increase vascular permeability, promoting tissue hydration and facilitating the migration of leukocytes into the cervical stroma [73]. PGs also enhance leukocyte recruitment by upregulating endothelial adhesion molecules, including intercellular adhesion molecule-1 (ICAM-1), which strengthen leukocyte–endothelial interactions and support inflammatory cell extravasation [44,73]. Additionally, PGs intensify the inflammatory milieu by downregulating secretory leukocyte protease inhibitor (SLPI), a key regulator of neutrophil protease activity. Reduced SLPI expression permits greater proteolytic activity of neutrophil-derived proteases (such as MMPs), contributing to ECM breakdown and facilitating cervical ripening [73,74].

Apart from their role in regulating inflammatory signaling, PGs directly influence cervical ECM remodeling. They increase the expression of hyaluronic acid-binding receptors on neutrophils, thereby supporting neutrophil activation and degranulation of neutrophil-secreted MMPs, which further enhances local inflammatory processes [75,76,77]. Furthermore, prostaglandins may directly stimulate the secretion of MMPs, which further enhances the enzymatic degradation of the cervical ECM [78,79,80]. Collectively, these findings position PGs as central integrators of inflammatory signaling and ECM remodeling, coordinating the molecular events that culminate in cervical softening and dilation.

### 3.4. Nitric Oxide

NO is another key mediator involved in the complex molecular network governing cervical ripening. This signaling molecule is generated through the activity of nitric oxide synthases (NOS), among which inducible nitric oxide synthase (iNOS) appears to play the predominant role in cervical remodeling [81]. Expression of iNOS increases markedly within the cervical stroma during the peripartum period, largely as a consequence of enhanced production by infiltrating macrophages and neutrophils. Substantial experimental evidence supports a critical role for NO in cervical ripening. Local administration of NOS inhibitors has been shown to delay and suppress cervical maturation, whereas administration of NO donors accelerates cervical remodeling [82,83,84,85]. Collectively, these findings identify NO as a crucial regulator of the cervical ripening process.

The effects of NO on cervical ripening biochemical pathways are mediated through both indirect and direct mechanisms. Indirectly, NO promotes local vasodilation, thereby facilitating tissue perfusion and the recruitment of inflammatory cells into the cervical stroma [86,87]. In addition, NO has been shown to stimulate the production of IL-8, a potent neutrophil chemoattractant, thereby enhancing chemotactic signaling within the local inflammatory microenvironment [88]. The increase in IL-8 concentration subsequently leads to enhanced chemotaxis of inflammatory cells and direct stimulation of MMPs activity in the cervical ECM. Through these interconnected actions, NO contributes to the establishment and maintenance of the inflammatory milieu characteristic of the ripening cervix.

Beyond its immunomodulatory effects, NO directly influences key biochemical pathways involved in cervical remodeling. Notably, NO enhances COX-2 activity, leading to increased prostaglandin synthesis within cervical tissues [89,90]. Given the central role of prostaglandins in ECM remodeling and leukocyte activation, NO-mediated upregulation of prostaglandin production represents an important mechanism through which NO promotes cervical remodeling. In addition to its effect on prostaglandin activity, NO also has a direct effect on MMPs, leading to an increase in their enzymatic activity [91]. Taken together, NO functions as a potent mediator linking inflammatory signaling and prostaglandin-dependent pathways, coordinating multiple processes that collectively drive cervical ripening.

### 3.5. Nuclear Factor-κB (NF-κB)

Beyond the factors that directly participate in the initiation and execution of the inflammatory response, particular attention should also be given to higher-order regulatory mechanisms. These upstream regulators are responsible for triggering, integrating, and coordinating the complex network of cellular and molecular events that collectively determine the onset, progression, and resolution of local inflammation.

Nuclear factor kappa B (NF-κB) is not a single molecule but a family of inducible and functionally interconnected transcription factors. This family comprises five members: NF-κB1 (p50), NF-κB2 (p52), RelA (p65), RelB, and c-Rel, which regulate the transcription of target genes through binding to specific κB enhancer sequences as homo- or heterodimeric complexes. Within the uterine cervix, NF-κB is increasingly recognized as a central regulatory node that coordinates inflammatory and tissue-remodeling pathways, thereby linking upstream signaling cascades with the downstream molecular and cellular events that drive cervical ripening process [92,93,94]. Under basal conditions, NF-κB is maintained in an inactive cytoplasmic state through binding to IκB inhibitory (inhibitor of Kappa-light-chain-enhancer in B cells) proteins [45]. Among the various IκB isoforms, IκBα plays a dominant role in restraining NF-κB signaling and is considered the key regulator of NF-κB activity [45].

Inflammatory cells, including neutrophils and macrophages, express pattern-recognition receptors (PRRs) that detect pathogen-associated molecular patterns (PAMPs) as well as damage-associated molecular patterns (DAMPs), endogenous molecules released from injured tissues and necrotic cells [95]. Activation of PRRs or stimulation by pro-inflammatory cytokines, represents a major upstream trigger of NF-κB signaling. In response to these inflammatory cues, the multi-subunit IκB kinase (IKK) complex becomes activated [96]. As the principal inhibitor of NF-κB, IκBα is tightly regulated by IKK, which phosphorylates IκBα and thereby initiates its ubiquitination and subsequent proteasomal degradation [96]. The loss of IκBα releases NF-κB from its inactive cytoplasmic complex, allowing its translocation to the nucleus. Once activated, nuclear NF-κB binds to specific κB regulatory elements within DNA and promotes the transcription of numerous genes involved in cervical remodeling, including pro-inflammatory cytokines (IL-1, IL-8, and IL-18), COX-2, iNOS, MMPs, components of the NLRP3 inflammasome, and various adhesion molecules [93,95,96,97]. Through the coordinated regulation of these downstream targets, NF-κB acts as a central transcriptional regulator that promotes ECM degradation and sustains the local inflammatory milieu characteristic of cervical ripening. Importantly, NF-κB contributes to cervical ripening through both indirect and direct mechanisms. In addition to amplifying the inflammatory cascade that drives cervical remodeling, NF-κB directly promotes ECM reorganization by upregulating the expression of MMPs, thereby facilitating collagen degradation and structural changes within the cervical stroma. Moreover, emerging evidence suggests that NF-κB is embedded within a self-perpetuating regulatory circuit. Activation of NF-κB promotes the production of inflammatory mediators (such as cytokines, PGs, NO and reactive oxygen species) that may subsequently reinforce NF-κB signaling, creating a positive feedback loop that sustains transcriptional activity and prolongs the inflammatory response [92,93,95,98]. This mechanism may be essential for coordinating and maintaining the molecular processes required for effective cervical ripening.

Collectively, the available evidence highlights NF-κB as a key upstream regulator of cervical ripening, acting at the intersection of inflammatory signaling and tissue remodeling to coordinate the complex molecular events that prepare the cervix for labor.

### 3.6. Mitogen-Activated Protein Kinases

Mitogen-activated protein kinases (MAPKs) constitute a family of proline-directed serine/threonine kinases that play a central role in intracellular signal transduction and are critically involved in macrophage-mediated inflammatory responses [99]. Among the MAPK family members, p38 MAPKs are particularly abundant in macrophages and have emerged as important regulators of inflammatory signaling [99]. Four p38 isoforms have been identified, namely p38α (MAPK14), p38β (MAPK11), p38γ (MAPK12/ERK6), and p38δ (MAPK13/SAPK4), with p38α representing the predominant subtype [100]. Activation of p38 MAPKs can be triggered by a wide range of stimuli, including ROS, pro-inflammatory cytokines, pathogens, growth factors, and estrogens, highlighting their role as key molecular sensors that integrate diverse extracellular signals into coordinated cellular responses [101]. Activation of MAPK signaling is initiated through phosphorylation by upstream MAPK kinases (MAPKKs), which trigger a cascade of sequential phosphorylation events leading to kinase activation. Among the MAPK pathways, the extracellular signal-regulated kinase (ERK) cascade, consisting of MEK1/2 and ERK1/2 (p42/p44), is one of the most extensively characterized. Following activation, MAPKs modulate the expression of numerous target genes by phosphorylating downstream transcription factors, including activator protein-1 (AP-1) and NF-κB (by promoting IκBα degradation) [100,102]. Through this mechanism, p38 MAPK signaling cascade promotes the transcription of key pro-inflammatory mediators, including cytokines (e.g., IL-1, IL-6 and IL-8), COX-2 and iNOS. A growing body of evidence suggests that, beyond its established role in systemic macrophage-mediated inflammation, p38 MAPK contributes significantly to the inflammatory signaling pathways associated with cervical ripening [102,103]. Importantly, p38 MAPK can further potentiate inflammatory signaling by promoting NF-κB activation and facilitating its translocation to the nucleus. Through this mechanism, p38 MAPK serves as an additional upstream stimulus that reinforces NF-κB-dependent transcription, thereby enhancing the expression of genes encoding inflammatory mediators within the cervical ECM [104]. The contribution of p38 MAPK to cervical ripening is also reflected in its ability to increase the expression of COX-2 and several pro-inflammatory cytokines, including IL-1, IL-6, and IL-8 [105]. Furthermore, p38 MAPK stimulates the expression of vascular cell adhesion molecule-1 (VCAM-1), which promotes leukocyte recruitment into cervical tissue and thereby supports the local inflammatory response [100]. In addition to its effects on inflammatory signaling, activated p38 MAPK directly contributes to cervical ECM remodeling by enhancing the synthesis of MMPs, particularly MMP-9, which has a great affinity toward cervical collagen covalent bonds [99,100,101,102].

Interestingly, beyond its well-established role in orchestrating local inflammatory responses within the cervical stroma, p38 MAPK appears to interact directly with components of the cervical ECM [106]. As discussed previously, HA and HS constitute the predominant GAG in the cervix during the peripartum period. Notably, HS has been shown to stimulate and sustain p38 MAPK activation, thereby promoting the transcription of genes encoding factors that actively participate in cervical remodeling [99,106,107]. Through its interaction with HS, p38 MAPK may participate in a bidirectional signaling network in which changes in ECM composition influence cellular responses, while activated cells simultaneously remodel the surrounding matrix. Sustained activation of p38 MAPK may consequently enhance the expression of pro-inflammatory cytokines, MMPs, and other mediators involved in ECM turnover, further facilitating the structural reorganization of cervical tissue. Such interactions highlight the multifaceted role of p38 MAPK in cervical ripening, positioning this kinase at the interface between inflammatory signaling and ECM remodeling processes.

### 3.7. NLRP3 Inflammasome

In addition to NF-κB and p38 MAPK signaling, the NLRP3 inflammasome has emerged as another important regulator of inflammatory processes associated with cervical ripening. Inflammasomes are intracellular multiprotein complexes that are assembled in response to cellular stress signals, including PAMPs and DAMPs [108]. The canonical inflammasome consists of three major components: a sensor molecule, such as NLRP3, the adaptor protein apoptosis-associated speck-like protein containing a caspase recruitment domain (ASC), and the effector enzyme pro-caspase-1 [108,109]. Within this complex, NLRP3 functions as a pattern-recognition receptor (PRR) capable of detecting a broad range of endogenous and exogenous signals [109]. Recognition of these stimuli initiates inflammasome assembly and promotes the activation of caspase-1, which subsequently processes the inactive precursors of IL-1β and IL-18 into their biologically active forms [109,110].

Beyond its role in cytokine maturation, activated caspase-1 can also cleave and activate gasdermin D (GSDMD), a pore-forming protein [111]. Cleavage of GSDMD results in the formation of membrane pores that facilitate the extracellular release of mature IL-1β and IL-18, thereby increasing their local bioavailability within the cervical microenvironment [111,112]. The concurrent release of cellular DAMPs further enhances inflammatory signaling in neighboring cells [113]. These mediators may subsequently activate neighboring cells and amplify local inflammatory signaling, thereby extending the effects of inflammasome activation beyond the initially stimulated cell population.

Once released, IL-1β and IL-18 act as potent mediators of inflammation and further amplify the inflammatory response [114,115]. Notably, IL-1β can itself function as a DAMP, thereby promoting additional NLRP3 activation and establishing a positive feedback mechanism that sustains inflammasome signaling. Increasing evidence indicates that activation of the NLRP3 inflammasome is closely associated with cervical ripening [113,116]. Elevated concentrations of IL-1β have consistently been observed within the cervical ECM during late pregnancy, while key inflammasome components, including ASC and caspase-1, have been identified in several maternal–fetal tissues, such as the fetal membranes, uterine body, and cervix at term [115,117,118]. Collectively, these observations support the concept that inflammasome assembly and activation represent integral elements of the cervical remodeling process preceding parturition.

The contribution of NLRP3 to cervical maturation appears to extend beyond cytokine production alone. Experimental studies have demonstrated that NLRP3-sufficient mice exhibit higher expression of hyaluronan synthase and proteoglycans genes than NLRP3-deficient animals, suggesting that NLRP3 signaling may directly participate in cervical stroma spatial reorganization [117]. In parallel, inflammasome-derived IL-1β stimulates the production of additional pro-inflammatory cytokines, enhances MMPs activity, and promotes prostaglandin synthesis while simultaneously reducing prostaglandin degradation. Through these mechanisms, IL-1β coordinates multiple aspects of the cervical remodeling cascade.

IL-18 appears to exert complementary effects by stimulating TNF-α production and, importantly, by supporting sustained nuclear translocation and activation of NF-κB [114,118]. This prolonged NF-κB signaling enhances the transcription of genes involved in the synthesis of NO, pro-inflammatory cytokines, and prostaglandins, thereby reinforcing the inflammatory microenvironment.

An additional layer of complexity may arise from the potential interaction between the NLRP3 inflammasome and p38 MAPK signaling. Experimental studies have demonstrated that LPS-induced activation of the NLRP3 inflammasome, accompanied by increased caspase-1 activity and elevated IL-1β production, is associated with enhanced p38 MAPK activation [119]. These findings suggest that NLRP3- and p38 MAPK-dependent signaling pathways are closely interconnected and may exhibit a considerable degree of functional interdependence. Given that p38 MAPK can be activated by a broad range of inflammatory mediators, including cytokines, it is plausible that NLRP3-driven cytokine release, particularly IL-1β secretion, contributes to the activation of p38 MAPK. Subsequent p38 MAPK signaling may then further amplify the local inflammatory response through the induction of additional pro-inflammatory mediators, thereby establishing a reinforcing regulatory network that promotes cervical inflammation and remodeling.

Taken together, these observations indicate that NLRP3, p38 MAPK, and NF-κB do not operate as isolated signaling pathways but rather form a highly interconnected regulatory network. Through extensive crosstalk and mutual amplification, these pathways coordinate inflammatory signaling, ECM remodeling, and immune cell recruitment, thereby ensuring the initiation, maintenance, and progression of cervical ripening.

### 3.8. High-Mobility Group Proteins (HMGB)

Another important regulator that appears to contribute to the proper progression of cervical ripening is high-mobility group box 1 (HMGB1), a member of the high mobility group (HMG) superfamily of non-histone chromatin-associated proteins [120]. The HMG family derives its name from the unusually high electrophoretic mobility of its members [120]. Among all HMG proteins, HMGB1 is the most abundant and has emerged as a multifunctional regulator involved in a wide range of cellular processes (especially inflammatory) [121]. Within the nucleus, HMGB1 acts as a DNA-binding factor that modulates gene transcription, participates in DNA repair, facilitates nucleosome assembly, and contributes to telomere maintenance [122]. However, the biological functions of HMGB1 are not restricted to the intracellular compartment. Under specific conditions, HMGB1 can be actively secreted or passively released into the extracellular environment, where it acquires a distinct signaling role [123].

The HMGB1 consists of two positively charged DNA binding domains—HMG A box and B box, a negatively charged C-terminal tail and N-terminal region [124]. The multifunctional properties of HMGB1 are closely linked to its unique structure and remarkable intracellular mobility. Within the nucleus, the A-box and B-box domains interact with DNA, whereas the acidic C-terminal tail associates with histones and contributes to chromatin organization [124,125]. Extracellular HMGB1 may originate from either active secretion or passive release. Active secretion occurs in immune cells in response to stimuli such as lipopolysaccharide (LPS), infections, and endogenous inflammatory signals, either directly or through vesicle-mediated pathways, including exosome release. Alternatively, HMGB1 can be passively released following cellular injury or cell death [123,124,125].

Extracellular HMGB1 functions as a DAMP molecule capable of interacting with several pattern-recognition receptors (PRRs), including the receptor for advanced glycation end products (RAGE) and Toll-like receptors (TLRs) [122,123,124,125]. Through these interactions, HMGB1 participates in the regulation of inflammatory and immune responses and serves as an important mediator of cell-to-cell communication during tissue remodeling [126]. Given that cervical ripening is characterized by tightly regulated inflammatory activation and extensive ECM remodeling, HMGB1 has attracted increasing attention as a potential upstream regulator of these processes. Accumulating evidence demonstrates that HMGB1 levels increase significantly within maternal–fetal tissues during the peripartum period, particularly in the cervix and amniotic fluid [127]. These findings suggest that HMGB1 may be significantly involved in the regulation of cervical ripening.

HMGB1 may contribute to cervical ripening through several interconnected mechanisms. One potential pathway involves its close functional interaction with the NLRP3 inflammasome. Following its release into the extracellular space, HMGB1 acts as a DAMP that promotes NLRP3 activation and subsequent IL-1β production, thereby amplifying local inflammatory signaling [128,129]. Furthermore, extracellular HMGB1 may promote the recruitment of inflammatory cells to sites of active tissue remodeling. Following its release, HMGB1 can directly interact with and activate the chemokine CXCL12, thereby enhancing chemotactic signaling and facilitating the migration of leukocytes into cervical tissue [130].

Another particularly intriguing aspect of HMGB1 biology is its close functional relationship with the NF-κB signaling pathway. Accumulating evidence suggests that HMGB1 can exert a potent stimulatory effect on NF-κB activity through several complementary mechanisms. First, HMGB1 has been shown to suppress the activity of IκBα, the principal inhibitory protein responsible for retaining NF-κB in the cytoplasm and preventing its nuclear translocation [131]. In addition, extracellular HMGB1 can interact with the receptor for advanced glycation end products (RAGE), which is expressed on inflammatory cells such as macrophages and neutrophils [96,121,132]. Activation of RAGE signaling promotes NF-κB activation and its subsequent translocation to the nucleus, where it stimulates the transcription of numerous genes involved in cervical remodeling and inflammation, including MMPs, COX-2, IL-1 and NO. Interestingly, the relationship between HMGB1 and NF-κB appears to be bidirectional. Activated NF-κB has been reported to enhance the extracellular release of HMGB1, thereby increasing the availability of this DAMP within the cervical microenvironment [120,133]. Extracellular HMGB1 can then further stimulate NF-κB signaling, creating a self-amplifying regulatory loop.

Beyond its effects on NLRP3 inflammasome activation and NF-κB signaling, HMGB1 appears to be closely linked to p38 MAPK activity, suggesting that this DAMP may coordinate multiple inflammatory pathways involved in cervical remodeling and ripening. Evidence suggests that the interaction between HMGB1 and RAGE receptor represents an important mechanism linking HMGB1 signaling to p38 MAPK activation [134,135]. RAGE is a transmembrane receptor expressed on the surface of various inflammatory cells, including macrophages and neutrophils [132]. Upon binding HMGB1, RAGE undergoes activation and initiates downstream signaling cascades involving p38 MAPK pathways (via MEK/ERK pathway). Activation of these pathways promotes the transcription of numerous pro-inflammatory mediators, including IL-1, COX-2, iNOS and MMPs [99]. Furthermore, as discussed previously, p38 MAPK can enhance NF-κB activity and support its sustained nuclear translocation, thereby further amplifying the expression of inflammation-related genes [104]. Through this signaling axis, HMGB1 may additionally indirectly strengthen NF-κB-dependent inflammatory responses and contribute to the maintenance of the pro-inflammatory microenvironment associated with cervical ripening.

Finally, in addition to its indirect effects on the local inflammatory response, HMGB1 may also contribute more directly to cervical remodeling. Experimental studies have demonstrated that exposure of fetal membranes to HMGB1 results in increased MMP-9 mRNA expression, suggesting that HMGB1 can stimulate the production of matrix-degrading proteases [136,137]. Given the pivotal role of MMP-9 in collagen degradation and ECM remodeling, these findings raise the possibility that HMGB1 participates directly in the structural changes that characterize cervical ripening, independently of its well-established pro-inflammatory actions.

Taken together, these findings suggest that HMGB1 functions as a key upstream regulator of cervical ripening, linking tissue stress signals with the activation of inflammatory and remodeling pathways. By coordinating the activity of multiple interconnected molecular networks, HMGB1 may play a pivotal role in orchestrating the transition of the cervix from a structurally stable tissue to one capable of supporting labor and delivery.

## 4. Papaverine Biochemistry

PPV is a naturally occurring benzylisoquinoline alkaloid classified as a phytochemical and is primarily isolated from *Papaver somniferum* (opium poppy), although it is also present in several other Papaver species [138]. Among more than 40 alkaloids identified in opium poppy, papaverine is one of the major constituents alongside morphine, codeine, noscapine, and thebaine [7]. Chemically, PPV (1-[(3,4-dimethoxyphenyl)methyl]-6,7-dimethoxyisoquinoline) is a benzylisoquinoline derivative and represents a distinct class of opium alkaloids lacking opioid receptor activity [139]. PPV is biosynthesized from two molecules of L-tyrosine through the benzylisoquinoline alkaloid pathway. Following condensation of dopamine and 4-hydroxyphenylacetaldehyde to form (S)-norcoclaurine, a series of hydroxylation, methylation, and oxidation reactions ultimately yields papaverine, with the (S)-reticuline pathway considered the predominant biosynthetic route in opium poppy [139,140].

PPV exerts its effects on the cells through several well-known mechanisms. From a biochemical point of view, PPV is classified as a phosphodiesterase (PDE) inhibitor [7]. PDEs have the ability to hydrolyze the 3′-phosphodiester bond in cyclic adenosine monophosphate (cAMP) and cyclic guanosine monophosphate (cGMP), converting them into their inactive forms, 5′-adenosine monophosphate and 5′-guanosine monophosphate, respectively [141,142,143,144]. By the blockage of the cAMP and cGMP phosphodiesterase, PPV leads to a great increase in cAMP and cGMP intracellular concentration [145,146].

Another, equally strong mechanism of action of PPV is the influence on the relaxation of smooth muscles [147,148]. First, PPV can directly inhibit myosin light chain kinase (MLCK), which then prevents the phosphorylation of the light chains of myosin heads. Then, the inactive myosin filaments cannot slide along the actin and cause muscle contraction [144]. Another equally important mechanism underlying the smooth muscle relaxant effect of PPV is its influence on intracellular calcium homeostasis. In addition to the direct inhibition of myosin light chain kinase (MLCK), PPV can inhibit voltage-dependent calcium channels, thereby reducing calcium influx into the cell while simultaneously promoting calcium removal from the cytoplasm [7,148]. The resulting decrease in intracellular Ca^2+^ concentration further suppresses the contractile activity of smooth muscle cells. Moreover, through phosphodiesterase inhibition and the consequent elevation of intracellular cAMP levels, PPV activates protein kinase A (PKA), which can stimulate potassium channels. The ensuing efflux of K^+^ ions leads to membrane hyperpolarization, reducing cellular excitability and contributing to smooth muscle relaxation [7,148,149].

The described effects of PPV seem to be well understood. It is also worth remembering that in addition to its direct effect, PPV may also have an indirect effect on a number of cellular processes [144]. It should be noted that both calcium ions, as well as cAMP and cGMP are among the most pivotal intracellular second messengers. These transmitters play an enormous regulatory role in a wide range of cellular pathways such as cell division, apoptosis, and development of local inflammatory response. Considering the fact that the mentioned substances are strongly involved in the whole process of parturition and cervical ripening, it seems reasonable to assume that PPV (via cAMP, cGMP and calcium ions) may have an indirect effect on the course of the molecular and biochemical pathways that contribute to these processes.

## 5. Papaverine and Cervical Ripening

At first glance, the role of PPV in cervical ripening may appear relatively straightforward and biologically plausible. As a non-selective phosphodiesterase inhibitor, PPV increases intracellular concentrations of cAMP and cGMP, promoting smooth muscle relaxation and modulating multiple signaling pathways involved in ECM remodeling, inflammation, and oxidative stress regulation. In obstetric practice, PPV has been associated with accelerated cervical dilatation and shortened labor duration, suggesting a potentially beneficial role in labor progression [6].

Despite these clinical observations, the molecular basis of PPV-induced facilitation of labor remains incompletely understood. Beyond its established effects on smooth muscle physiology, accumulating evidence indicates that PPV can influence numerous biological processes implicated in cervical remodeling, including inflammatory signaling, nitric oxide pathways, oxidative stress responses, and ECM homeostasis. Notably, several molecular pathways reportedly modulated by PPV overlap with those considered central to cervical ripening. However, the biological consequences of these interactions remain unclear, as some reported effects appear difficult to reconcile with the current inflammatory model of cervical ripening. Therefore, a detailed examination of the interactions between PPV and key mediators of cervical ripening may provide valuable insight into the mechanisms underlying its clinical effects during labor.

PPV may modulate local inflammatory processes involved in cervical ripening through several intracellular signaling pathways, with p38 MAPK representing one of its key molecular targets. Experimental evidence suggests that PPV may directly inhibit p38 MAPK activation while simultaneously attenuating upstream signaling through the MEK/ERK cascade, thereby exerting broader regulatory effects on inflammatory signal transduction [150,151,152,153]. Inhibition of p38 MAPK signaling may have several important downstream consequences. First, p38 MAPK suppression may attenuate activation of NF-κB, a central regulator of inflammatory gene expression. Consequently, NF-κB-dependent transcription of numerous pro-inflammatory mediators, including COX-2, iNOS, IL-1, IL-6 may be reduced. Importantly, p38 MAPK itself directly regulates the transcription of many of these inflammatory genes (such as COX-2 and IL-1). Therefore, PPV-mediated inhibition of p38 MAPK may further suppress the expression of pro-inflammatory mediators through both NF-κB-dependent and NF-κB-independent mechanisms. Furthermore, inhibition of p38 MAPK may be associated with decreased expression of VCAM-1, potentially limiting leukocyte recruitment and infiltration into cervical tissue. In addition, p38 MAPK inhibition may reduce the synthesis and secretion of neutrophilic MMP-9, thereby attenuating cervical ECM remodeling and collagen cross-link degradation. Collectively, these observations suggest that PPV may exert broad anti-inflammatory and anti-remodeling effects on several molecular pathways considered important for physiological cervical maturation.

Beyond its indirect effects mediated through p38 MAPK inhibition, PPV may also directly suppress NF-κB signaling, further amplifying its anti-inflammatory potential. Current evidence suggests that PPV may exert a dual inhibitory effect on NF-κB activity through both upstream signaling modulation and direct interference with NF-κB activation [154]. First, PPV may attenuate NF-κB activation by suppressing receptor for advanced glycation end products (RAGE)-dependent signaling [155,156]. Inhibition of RAGE reduces the transduction of pro-inflammatory signals that normally converge on NF-κB, thereby limiting its activation. Second, phosphodiesterase inhibition by PPV leads to intracellular accumulation of cAMP, which has been shown to directly impair the nuclear translocation of NF-κB and consequently reduce its transcriptional activity [154,155,157,158]. As a result, PPV may suppress the expression of numerous NF-κB-dependent genes involved in the initiation and propagation of inflammatory responses. Importantly, NF-κB occupies a pivotal position within the inflammatory network governing cervical ripening, integrating signals from cytokines, DAMPs and multiple intracellular signaling pathways. Therefore, suppression of NF-κB activity may result in substantial attenuation of the local inflammatory response within cervical tissue. Moreover, as p38 MAPK is known to positively regulate NF-κB activation, the inhibitory effect of papaverine on p38 MAPK may provide an additional mechanism contributing to NF-κB suppression, further reinforcing its overall anti-inflammatory activity.

Remaining within the context of local inflammatory regulation, a particularly interesting interaction exists between PPV and high-mobility group box 1. Experimental evidence suggests that PPV may act as a competitive antagonist of the HMGB1–RAGE axis by mimicking the RAGE-binding domain of HMGB1 [156,159]. As a result, PPV may interfere with HMGB1–RAGE interactions, leading to suppression of downstream NF-κB and p38 MAPK signaling. In turn, inhibition of these pathways may reduce the transcription of numerous pro-inflammatory genes involved in cervical inflammation and remodeling, including COX-2, iNOS, IL-1, and IL-6.

**Figure 3:**
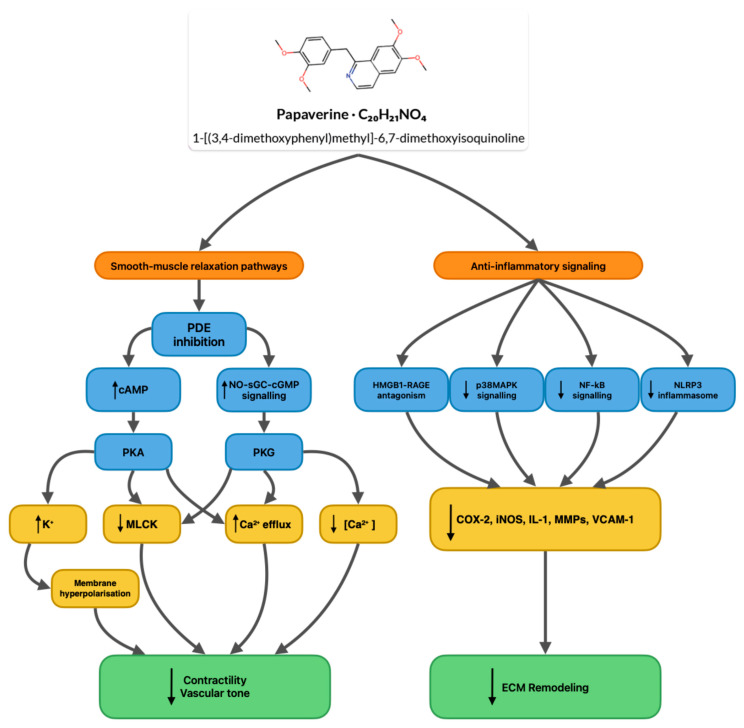
Proposed mechanisms of PPV action relevant to cervical ripening. Papaverine exerts both smooth-muscle relaxant and anti-inflammatory effects through multiple molecular pathways. Phosphodiesterase inhibition increases intracellular cyclic nucleotide signaling via the cAMP–PKA pathway and potentiates NO–sGC–cGMP–PKG signaling by preventing cGMP degradation, thereby prolonging the downstream biological effects of NO. These mechanisms promote smooth-muscle relaxation through reduced intracellular Ca^2+^ availability, decreased MLCK activity and membrane hyperpolarization. Simultaneously, PPV suppresses several inflammatory signaling pathways, including HMGB1–RAGE, NF-κB, p38 MAPK, and the NLRP3 inflammasome, resulting in reduced expression of inflammatory mediators associated with ECM remodeling.

Another potentially important mechanism through which PPV may modulate cervical inflammation involves the NLRP3 inflammasome. Emerging evidence suggests that PPV may suppress NLRP3 inflammasome activation, thereby limiting the processing and maturation of IL-1β, one of the principal cytokines implicated in cervical ripening [160]. Attenuation of NLRP3 activity may have several downstream consequences relevant to cervical remodeling. Reduced inflammasome activity may be associated with decreased expression of hyaluronan synthase and proteoglycans genes, potentially impairing cervical cytoarchitectural remodeling [117]. Given the critical role of hyaluronan in tissue hydration and cervical softening, suppression of this pathway may adversely affect physiological cervical ripening. Reduced IL-1β production may decrease the expression and secretion of MMPs. Furthermore, diminished IL-1β signaling may lead to reduced COX-2 expression and relief of prostaglandin dehydrogenase inhibition, thereby promoting prostaglandin degradation and further limiting inflammatory signaling. As IL-1β itself can act as a danger-associated molecular signal capable of amplifying inflammasome activity, its reduction may also attenuate further NLRP3 activation, creating a negative feedback loop that suppresses local inflammation. Collectively, these effects may contribute to the inhibition of multiple signaling pathways involved in cervical inflammatory remodeling and maturation. A plausible explanation for this phenomenon may be related to the phosphodiesterase-inhibitory activity of PPV. By increasing intracellular cAMP levels, PPV may promote cAMP binding to NLRP3, triggering its ubiquitination and subsequent degradation in macrophages [161,162]. Through this mechanism, PPV may directly interfere with inflammasome activation and further attenuate local inflammatory signaling during cervical ripening (Figure 3).

Taken together, the available experimental evidence suggests that PPV exerts predominantly anti-inflammatory effects through simultaneous modulation of several interconnected signaling pathways, including p38 MAPK, NF-κB, HMGB1, and NLRP3 inflammasome. It should be noted that the immunomodulatory effects of papaverine may vary depending on the experimental model, cell type, and biological context. Nevertheless, the evidence currently available in the context of cervical ripening predominantly supports an anti-inflammatory molecular profile. The convergence of these effects on key mediators of cervical inflammation and ECM remodeling raises the possibility that PPV may attenuate multiple molecular processes traditionally considered essential for physiological cervical ripening.

## 6. Discussion

The findings summarized in this review reveal an apparent discrepancy between the reported molecular effects of PPV and its observed clinical efficacy during labor. Experimental evidence suggests that PPV may suppress several signaling pathways considered central to cervical ripening, including p38 MAPK, NF-κB, HMGB1/RAGE, NLRP3 inflammasome signaling, and downstream inflammatory mediators such as IL-1β, IL-6, COX-2, prostaglandins, and MMPs. Collectively, these observations would theoretically be expected to attenuate rather than promote cervical remodeling. However, clinical studies reported that PPV improved the Bishop score and shortened the catheter balloon insertion-to-delivery interval [6], whereas a second randomized trial, in which PPV was administered at oxytocin initiation, found no reduction in the time to active labor or in the catheter-to-delivery interval but a lower rate of cesarean delivery [163]. Notably, these clinical observations do not necessarily demonstrate accelerated biochemical cervical ripening. Rather, they may reflect improved cervical compliance resulting from altered tissue biomechanics and smooth muscle relaxation. Additionally, the differences between these studies should be interpreted with caution, as they may reflect substantial heterogeneity in patient characteristics, baseline cervical status, parity, timing of papaverine administration, concomitant oxytocin use, induction protocols, and study design rather than true differences in drug efficacy.

Importantly, the mechanistic evidence discussed in this review is heterogeneous and should be interpreted accordingly. While some pathways involved in cervical ripening have been investigated directly in human cervical tissue, a substantial proportion of the available data derives from animal models, in vitro experiments, or studies performed in non-cervical tissues. Moreover, many of the reported effects of PPV reflect modulation of upstream signaling pathways rather than direct effects on the terminal effector mechanisms responsible for ECM remodeling and cervical softening [6,163]. In contrast, clinical studies evaluate the integrated physiological response of the cervix in vivo, where biomechanical, endocrine, immunological, and contractile processes interact simultaneously. This disparity between reductionist experimental models and the complex physiological environment of human labor may explain the apparent discrepancy between the PPV molecular profile and its reported clinical effects. Accordingly, the mechanistic framework proposed in this review should be regarded as hypothesis-generating and requires validation in human cervical tissue and adequately powered clinical studies.

One possible explanation for this discrepancy may involve NO signaling. Although PPV may reduce inflammatory NO production through inhibition of NF-κB- and p38 MAPK-dependent iNOS expression, its phosphodiesterase-inhibitory activity simultaneously promotes intracellular cGMP accumulation, thereby potentiating downstream NO signaling. Under physiological conditions, NO activates soluble guanylate cyclase (sGC), resulting in increased cGMP production and subsequent activation of protein kinase G (PKG). PKG promotes smooth muscle relaxation by reducing intracellular calcium availability and decreasing the calcium sensitivity of the contractile apparatus through modulation of myosin light-chain phosphorylation [164,165]. Consequently, inhibition of cGMP degradation by PPV may amplify and prolong the biological effects of NO, even in the absence of increased NO synthesis. Enhanced NO–cGMP signaling may therefore increase tissue compliance, promote smooth muscle relaxation, and improve cervical distensibility independently of classical inflammatory remodeling pathways (Figure 3). In addition, NO-mediated vasodilation may improve local tissue perfusion and microvascular blood flow within the cervix. Although the precise implications of this effect remain unclear, enhanced microcirculation could facilitate the delivery of inflammatory mediators and support leukocyte trafficking into cervical tissue during cervical ripening.

Recent advances in cervical biology provide further support for the hypothesis that PPV may facilitate labor through mechanisms extending beyond classical inflammatory remodeling. Traditionally, the cervix has been viewed primarily as a connective tissue structure composed predominantly of ECM components. However, evidence suggests a more complex functional organization. While the external cervical os consists largely of collagen-rich connective tissue containing only sparse smooth muscle cells, the internal cervical os contains a substantial smooth muscle component arranged in circumferential, sphincter-like patterns [9,13,20]. These observations have led to the proposal that the internal os may function as an active smooth muscle sphincter involved in maintaining cervical competence during pregnancy and regulating cervical opening during labor [13]. In this context, the pharmacological properties of PPV may be particularly relevant. Through phosphodiesterase inhibition and subsequent augmentation of cAMP- and cGMP-dependent signaling, PPV promotes smooth muscle relaxation and reduces contractile activity. If the internal cervical os functions as a smooth muscle sphincter, PPV-induced relaxation of this structure could directly reduce cervical resistance and facilitate dilatation independently of ECM remodeling. Such a mechanism could potentially explain why PPV demonstrates clinically beneficial effects on labor progression despite its predominantly inhibitory influence on several inflammatory pathways implicated in cervical ripening.

Additional factors potentially contributing to the clinical efficacy of PPV may involve PG signaling. Although PGs are widely recognized as key mediators of cervical ripening and labor, their biological effects depend not only on local PGs concentrations but also on the expression profile of their receptors and downstream intracellular signaling pathways [68]. Importantly, pregnancy and labor are associated with dynamic changes in prostaglandin receptor distribution within the myometrium. Notably, studies have demonstrated higher myometrial expression of the EP1 and EP3 receptors in the uterine fundus at term compared with the lower uterine segment [66,67,68,69]. This shift in receptor distribution may suggest a relative reduction in contractile prostaglandin signaling within the lower uterine segment, potentially allowing the relaxatory effects mediated by EP2 receptors to become more prominent. Mechanistically, EP2 signaling is coupled to adenylate cyclase activation and subsequent intracellular cAMP accumulation, whereas EP1 is coupled to Gq-dependent signaling pathways that promote intracellular calcium mobilization and smooth muscle contraction. Consequently, the prostanoid-receptor balance in the lower uterine segment shifts toward EP2-mediated, relaxation-promoting signaling at term. In this context, phosphodiesterase inhibition by PPV may further augment EP2-mediated signaling by preventing cAMP degradation and prolonging its intracellular effects. Consequently, PPV could potentiate PGs-induced relaxation of the lower uterine segment and cervical region, thereby facilitating cervical dilatation despite its predominantly inhibitory influence on inflammatory pathways implicated in cervical remodeling. It should be noted, however, that this EP2-based mechanism is inferred from the lower uterine segment myometrium rather than from cervical stroma, where prostaglandin-induced remodeling is mediated predominantly by EP4; direct evidence for an EP2–cAMP action of PPV in human cervical tissue is currently lacking. Together with the NO–cGMP pathway and the proposed sphincter-like organization of the internal cervical os, these observations suggest that PPV may facilitate labor progression and cervical ripening through modulation of tissue biomechanics and smooth muscle function. Consequently, the net clinical effect of PPV may be determined not only by its influence on inflammatory remodeling, but also by its ability to reduce cervical resistance and enhance tissue compliance, potentially outweighing its inhibitory effects on inflammatory signaling pathways.

Taken together, the available evidence suggests that the effects of PPV on cervical ripening cannot be fully explained by the currently recognized inflammatory pathways alone. Although PPV appears to suppress multiple molecular processes traditionally considered essential for cervical ripening, including cytokine production, inflammasome activation, prostaglandin synthesis, and ECM remodeling, the available clinical evidence nevertheless suggests that PPV may facilitate cervical dilatation. This apparent paradox supports the hypothesis that PPV primarily enhances cervical compliance through biomechanical mechanisms rather than by directly promoting ECM remodeling. In particular, relaxation of the smooth muscle-rich internal cervical os, together with modulation of NO–cGMP signaling and prostanoid receptor pathways, may reduce cervical resistance during labor. These observations do not contradict the established role of inflammatory reaction in cervical ripening but rather suggest that complementary biomechanical mechanisms may also contribute to cervical softening and dilation during labor. Functional biomechanical mechanisms may represent an additional, underappreciated determinant of labor progression. Further studies performed directly in human cervical tissue are required to determine how these mechanisms interact and to better define the role of PPV in labor progression.

## 7. Conclusions

The available evidence suggests that the clinical effects of PPV may be explained more convincingly by modulation of cervical biomechanics than by direct enhancement of cervical remodeling. By promoting smooth muscle relaxation and reducing functional resistance within the lower uterine segment and internal cervical os, PPV may facilitate cervical dilatation despite its predominantly anti-inflammatory molecular profile. Although this hypothesis requires validation in studies performed directly in human cervical tissue, it provides a plausible mechanistic framework that may help reconcile the apparent discrepancy between the molecular and clinical effects of papaverine. Future studies combining mechanistic investigations in human cervical tissue with adequately powered randomized clinical trials will be essential to validate this hypothesis and determine the optimal role of papaverine in modern obstetric practice.

## Data Availability

No new data were created or analyzed in this study. Data sharing is not applicable to this article.

## Abbreviations

The following abbreviations are used in this manuscript:

cAMP	cyclic adenosine monophosphate
cGMP	cyclic guanosine monophosphate
COX-2	cyclooxygenase-2
CXCL-12	C-X-C motif chemokine ligand 12
DAMP	damage-associated molecular pattern
ECM	extracellular matrix
EP2	prostaglandin E2 receptor subtype 2
HMGB1	high-mobility group box 1
ICAM-1	intercellular adhesion molecule 1
IL	interleukin
iNOS	inducible nitric oxide synthase
MAPK	mitogen-activated protein kinase
MLCK	myosin light-chain kinase
MMP	matrix metalloproteinase
NF-κB	nuclear factor κB
NLRP3	NOD-like receptor protein 3
NO	nitric oxide
PAMP	pathogen-associated molecular pattern
PDE	phosphodiesterase
PKA	protein kinase A
PKG	protein kinase G
PPV	papaverine
RAGE	receptor for advanced glycation end-products
RCT	randomized controlled trial
TIMP	tissue inhibitor of metalloproteinases
